# Dopamine Increases Accuracy and Lengthens Deliberation Time in Explicit Motor Skill Learning

**DOI:** 10.1523/ENEURO.0360-23.2023

**Published:** 2024-01-16

**Authors:** Li-Ann Leow, Lena Bernheine, Timothy J. Carroll, Paul E. Dux, Hannah L. Filmer

**Affiliations:** ^1^School of Psychology, The University of Queensland, St Lucia, 4072, Australia; ^2^Centre for Sensorimotor Performance, School of Human Movement & Nutrition Sciences, St Lucia, 4067, Australia; ^3^School of Sport Science Faculty of Sport Governance and Event Management, University of Bayreuth, 95447 Bayreuth, Germany

**Keywords:** dopamine, motor control, motor performance, motor skill learning, reward, task error

## Abstract

Although animal research implicates a central role for dopamine in motor skill learning, a direct causal link has yet to be established in neurotypical humans. Here, we tested if a pharmacological manipulation of dopamine alters motor learning, using a paradigm which engaged explicit, goal-directed strategies. Participants (27 females; 11 males; aged 18–29 years) first consumed either 100 mg of levodopa (*n* = 19), a dopamine precursor that increases dopamine availability, or placebo (*n* = 19). Then, during training, participants learnt the explicit strategy of aiming away from presented targets by instructed angles of varying sizes. Targets jumped mid-movement by the instructed aiming angle. Task success was thus contingent upon aiming accuracy and not speed. The effect of the dopamine manipulations on skill learning was assessed during training and after an overnight follow-up. Increasing dopamine availability at training improved aiming accuracy and lengthened reaction times, particularly for larger, more difficult aiming angles, both at training and, importantly, at follow-up, despite prominent session-by-session performance improvements in both accuracy and speed. Exogenous dopamine thus seems to result in a learnt, persistent propensity to better adhere to task goals. Results support the proposal that dopamine is important in engagement of instrumental motivation to optimize adherence to task goals, particularly when learning to execute goal-directed strategies in motor skill learning.

## Significance Statement

While animal studies show a central role of dopamine in skill learning, such evidence is lacking in neurotypical humans. We provide evidence for a role of dopamine in learning explicit, goal-directed motor strategies in neurotypical humans. Exogenous dopamine at training improved accuracy, as participants traded speed for accuracy in a task that focused on accuracy, not speed. Importantly, this behavior persisted at a no-drug follow-up, suggesting that dopamine resulted in a learnt, persistent propensity to better adhere to task goals. Dopamine influences instrumental motivation to optimize adherence to task goals in motor learning, not only influencing performance at initial learning but also when retrieving such strategies to solve familiar motor problems.

## Introduction

Motor skill learning is essential for survival: a hungry bear catching salmon must adapt its paw-strikes to dynamic task parameters, such as the movement and friction of the salmon's body and the forces applied by river waters. Reward is a potent modulator of skilled movement, affecting movement in two primary ways. First, rewards can increase the vigor of movements (Manohar et al., 2015; Carroll et al., 2019; Codol et al., 2019, for a review, see Shadmehr et al. (2019)) even when rewards are not performance contingent (Takikawa et al., 2002). Second, rewards can boost skill learning. For example, rewarding participants for achieving performance criteria can speed up learning (Vassiliadis et al., 2021) or improve retention of motor learning (Abe et al., 2011; Madelain et al., 2011; Galea et al., 2015). The potency of reward in altering behavior and learning is perhaps unsurprising, as animals must learn motor skills to attain resources for survival (Barron et al., 2010).

Experimental manipulations of reward do not, however, always benefit motor learning (Steel et al., 2016; Spampinato et al., 2019). This is perhaps because rewarding participants for achieving some experimenter-determined performance criterion does not directly alter a key learning signal: success or failure at achieving the task goal. Failures to achieve task goals, termed task errors, seem essential to forming memories that improve future motor performance, and such memories take at least two forms: deliberative goal-oriented strategies and automatic stimulus–response associations (Leow et al., 2020).

Task errors can be conceptualized as a form of reward prediction error (Leow et al., 2020), which is a broader term describing discrepancies between predicted rewards and received rewards (Sutton and Barto, 1998). Transient bursts of firing by midbrain dopamine neurons triggered by reward prediction errors have been thought to “stamp-in” associations between a stimulus and its associated response, resulting in the formation of automatic stimulus–response mappings. Increasing evidence also implicates dopamine in the engagement of deliberative, goal-oriented behaviors (Akam and Walton, 2021). Dopamine might therefore play dual roles in skill learning: stamping in stimulus–response associations to facilitate automaticity with training and motivating the engagement of goal-oriented strategies.

While nonhuman animal studies implicate dopamine-dependent circuits in skill learning (Hosp et al., 2011), inter-species differences in the cognitive and neural processes associated with the dopamine system (Khan et al., 1998) limit generalization of findings from animals to humans. Evidence for the role of dopamine in human motor learning comes from studying how dopamine medications alter learning in patients with impaired dopamine function (Kwak et al., 2010), but heterogeneity in disease phenotypes complicates inferences from such work (Cools et al., 2022). Causal evidence for the role of dopamine in skill learning in neurotypical humans is scarce and limited to studies of elementary motor tasks such as repeating simple thumb-abduction movements (Floel et al., 2008; Flöel et al., 2008; Rosser et al., 2008) or tracking on-screen targets (Chen et al., 2020a). Of import, some studies have found no effect of manipulating dopamine on motor learning (Quattrocchi et al., 2018; Palidis et al., 2021). These null results might have resulted from paradigms which predominantly engage implicit learning (Palidis et al., 2021), or which do not dissociate effects of explicit and implicit learning processes (Quattrocchi et al., 2018), which can have mutually compensatory effects on behavior (Albert et al., 2022). Indeed, in reinforcement learning tasks, explicit, goal-directed processes can be more sensitive to effects of dopamine manipulations than implicit, automatic processes (Sharp et al., 2016). Similarly, explicit processes are more sensitive to reward manipulations than implicit processes in motor learning (Codol et al., 2018; Holland et al., 2018). Manipulations of dopamine and reward might thus be more observable in learning tasks driven by explicit knowledge of the task structure and strategies to achieve task goals.

Here, we investigated the role of dopamine in learning goal-directed motor strategies. After consuming the dopamine precursor levodopa or placebo, participants learnt a strategy of aiming away from presented targets by angles of varying sizes (Georgopoulos and Massey, 1987). Participants had to employ strategic aiming to hit targets, as targets would jump mid-movement by the instructed aiming angle. Thus, task success (i.e., target-hitting) was contingent upon successful aiming. Effects of the dopamine manipulations were assessed during training and at a no-drug follow-up session.

## Materials and Methods

### Participants

Thirty-eight neurotypical young adults (27 females; median age, 21.58 years; range, 18–29 years) were recruited from The University of Queensland community and were reimbursed for participation (AUD$20/h). Participants were screened for neurological and psychiatric conditions and contraindications for levodopa and provided written informed consent. In accordance with the National Health and Medical Research Council's guidelines, this experiment was approved by the human ethics committee at The University of Queensland. No datasets were excluded from the study.

As no published studies on the effects of levodopa on explicit motor skill learning in neurotypical adults existed at the time of planning the study, our sample size was guided by previous studies that examined effects of levodopa on tasks requiring cognitive effort (Vo et al., 2017); however, we did not explicitly conduct a power analysis based on these studies, as their design was dissimilar to ours. Half of the participants (*n* = 19) were randomly assigned to the levodopa condition (16 females; median age, 20.74 years; range, 18–25 years), and the other half were assigned to the placebo condition (*n* = 19; 11 females; median age, 22.42 years; range, 19–29 years).

### Drug manipulation

The levodopa group consumed the dopamine precursor, a Madopar 125 (levodopa 100 mg/benserazide 25 mg) tablet, whereas the placebo group consumed a crushed multivitamin (Centrum for women). Double blinding was achieved by having an experimenter who was not involved in data collection crush and disperse the tablets in orange juice before consumption by the participant. Behavioral testing of the aiming task began ∼60 min after tablet administration, around the time of peak plasma concentration (Contin and Martinelli, 2010). The drug manipulation was only employed on the training session, and not at follow-up.

### Apparatus

A vBot planar robotic manipulandum was used (Howard et al., 2009). This apparatus has a low-mass, two-link carbon fiber arm and measures position with optical encoders sampled at 1,000 Hz. Participants completed the aiming task while seated on a height-adjustable chair at their ideal height for viewing the screen. Visual feedback was presented on a horizontal plane on a 27″ LCD computer monitor (ASUS, VG278H, set at 60 Hz refresh rate) mounted above the vBot and projected to the subject via a mirror in a darkened room. Participants had no direct vision of their hand. Hand position was represented by a red cursor (0.25-cm-radius circle) on a black background. The mirror also allowed visual feedback of the target (a 0.5-cm-radius circle) and the start location (a 0.35-cm-radius circle), which was aligned 10 cm to the right of the participant's midsagittal plane at approximately mid-sternum level.

### Procedure

Participants were first given the following instructions: “You are about to perform sets of 80 trials in which you will be asked to move at specific angle relative to the target in clockwise direction. The angle will differ between blocks. We will tell you what the angle is for each block. First, make up your mind and then try to keep your movement straight and fast. Try your best to execute your movements as accurately as possible and hit as many targets as possible. Between each set we will ask you to aim towards the target in a 0° angle, that means you should hit the target directly. These sets will contain 20 trials.” Instructions were followed by a demonstration by the experimenter on how to re-aim by 90° away from the presented target.

**Figure 1.**
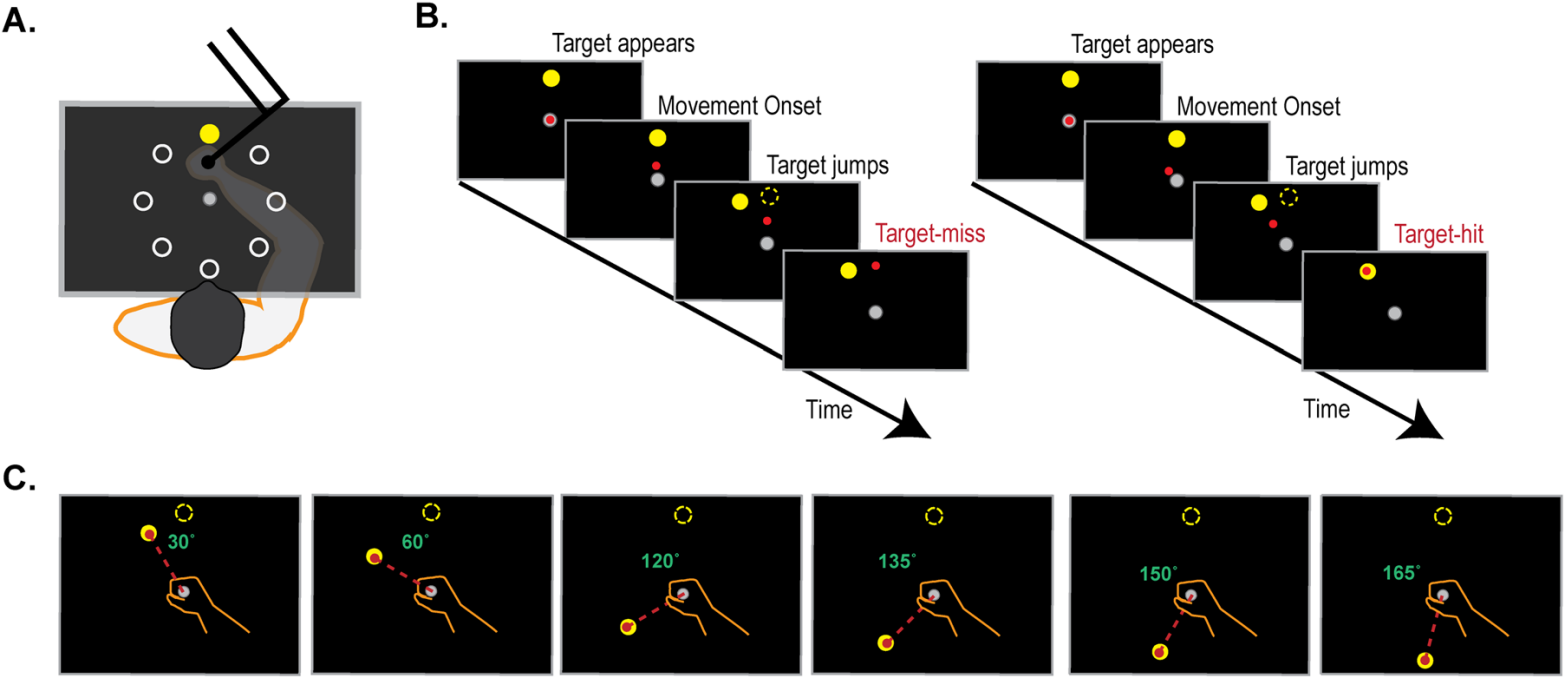
***A***, Participants used a robotic manipulandum to move an on-screen cursor from a central start circle to one of 8 possible target locations (0°, 45°…315°): target order was randomized within each bin of eight trials such that each target appeared once within each bin. No direct visual feedback of the hand was available. Online movement corrections were disincentivized by only making feedback of hand position available for the last 4 cm of the 9 cm start-target distance. Feedback of hand position was also removed as soon the 9 cm start-target distance was achieved. ***B***, Trial sequence for the aiming trials. Before the start of each aiming block (where each block = 10 bins, or 80 trials), participants were instructed to aim away from the presented target at angles of varying sizes (0°, 30°, 60°, 120°, 135°, 150°, 165°; ***C***). In each aiming trial, participants saw a target that jumped mid-movement (4 cm into the 9 cm start-target distance) by the instructed aiming angle relative to the target. As the target jump was not linked to sensory properties of the participant movements, the target jump did not result in sensory prediction errors (i.e., discrepancies between predicted and actual sensory outcomes of movements). Instead, the target-jump results in task errors, or failures to achieve the task goal of hitting the target, if the participant fails to aim by the instructed angle.

Participants were given seven aiming blocks. In each block, participants were asked to re-aim away from the presented target by one of the following angles, 0°, 30°, 60°, 120°, 135°, 150°, and 165°, similar to previous work (Georgopoulos and Massey, 1987; Pellizzer and Georgopoulos, 1993; Bhat and Sanes, 1998; Neely and Heath, 2010; McNeely and Earhart, 2012), where block order was randomized between subjects. Each block contained 10 bins of eight trials each, making a total of 80 trials per block. Each bin contained one presentation of all eight possible target locations (0°, 45°, 90°… 315°), in random order within each bin. All trials commenced by displaying the central start circle, with participants then moving the cursor within 1 cm of the start circle. If participants did not move the cursor to the start after 1 s, the robotic manipulandum moved the participant's cursor to the start circle, using a simulated two-dimensional spring with the spring constant magnitude linearly increasing over time. After the cursor remained within the start circle at a speed below 0.1 cm/s for 200 ms, targets appeared in random order at one of eight locations 9 cm away from the start circle (0°, 45°, 90°… 315°; Fig. 1*A*).

In trials contained within each aiming block, the target “jumped” mid-movement (i.e., when movement extent reached 4 cm into the 9 cm start-target distance): the size of the target-jump was the same as the instructed aiming angle, such that successful aiming was necessary to attain the task goal of hitting the target (Leow et al., 2020). Target “jumps” were achieved by extinguishing the target and re-displaying the target at the new location as soon as movement extent exceeded 4 cm out of the 9 cm start-target distance (Fig. 1*B*). Each aiming block was followed by a 20-trial washout block, where the task was to move straight to the presented targets: no target-jumps occurred during these washout blocks. Across all trials, cursor feedback was only given after movement extent exceeded 4 cm and was then extinguished after movement extent exceeded the 9 cm start-target distance. We have previously found this effective in disincentivizing online movement corrections (Leow et al., 2018, 2020, 2021). To economize study duration, a washout block was not provided after the 0° block or the final re-aiming block. Across all trials, including the washout blocks, auditory feedback in the form of a beep sound (coin.wav from Super Mario) was given when the reach angle measured at 4 cm into the 9 cm start-target distance reached an accuracy criterion of being within a +/10° range of the ideal aiming angle. This auditory feedback was implemented to maintain participant engagement in the long test session.

After an overnight delay, participants returned for a follow-up session, scheduled a minimum of 18 h after the first session. The task was identical to the first session. On the second session, all participants consumed a placebo pill dispersed in orange juice. Participant blinding was assessed after both sessions.

### Additional assessments

To assess for changes in arousal, heart rate, blood pressure, and mood, we measured these variables at the beginning, after 1 h, and at the end of each session (Vo et al., 2016). Mood was assessed using the Mood Rating Scale (Bond and Lader, 1974) which includes 16 items separated in the following factors: alertness, contentedness, and calmness. The participants could rate each element within a range of 10 points. The three factors were evaluated as a total score.

Individual differences in baseline dopamine function affects responsivity to dopamine medications. Individual dopamine baseline function is partly predicted by impulsivity and working memory. Impulsivity, as measured by the behavioral inhibition scale (BIS-11), is associated with dopamine D2/D3 receptor availability in the midbrain (Buckholtz et al., 2010). BIS scores can predict effects of methylphenidate on learning (Clatworthy et al., 2009). Similarly, working memory span has been associated with dopamine synthesis capacity, with a medium to large effect size for the correlation between listening span scores and dopamine synthesis capacity (Cools et al., 2008). Working memory span can predict effects of pharmacological manipulations of dopamine (Broadway et al., 2018; Fallon et al., 2019). To account for individual differences in baseline dopamine function, we used scores on assessments of impulsivity and working memory capacity as covariates. Working memory was measured with the memory updating task from a validated working memory battery (Lewandowsky et al., 2010). The memory updating task had participants remember a set of digits presented for 1 s in separate on-screen locations and update these digits through arithmetic operations shown on-screen for 1.3 s at corresponding locations. The number of digits presented (i.e., set size) varied from three to five across trials. After a varying number of update operations (between two and six), question mark prompts appeared in each frame. There was no time limit for the recall, and there was no performance feedback. The operations ranged from +7 to −7, excluding 0, and the results from 1 to 9. There were 15 trials in total and two practice trials. Performance on the memory updating task was assessed both at training and at follow-up, but to avoid the influence of practice effects, we used memory updating data from only the training session for analysis.

### Data analysis

Our task instructions to “try your best to execute your movements as accurately as possible” and target-jump manipulations (i.e., where to target shifted mid-movement at the required aiming angle, such that target-hitting required accurate aiming) emphasized accuracy of aiming movements, rather than the speed of reaction times nor the speed of the executed movements. Thus, our primary dependent variable of interest was accuracy of aiming at the instructed angle. Aiming accuracy was estimated from the directional error between the ideal aiming angle and the absolute values of the actual reach direction, where reach direction was measured at 20% of the start-target distance (i.e., 1.8 cm into the 9 cm start-target distance).

While our instructions and task structure emphasized aiming accuracy, we were also interested in evaluating changes in the speed of movement planning and movement execution. To this end, we quantified the following: (1) reaction time, defined as the interval from target appearance to movement onset, where movement onset was the time at which the hand speed first exceeded 2 cm/s, and (2) peak velocity. Furthermore, as previous research has implicated reward in movement precision (Manohar et al., 2015), we also estimated precision of aiming by quantifying error variability, defined as the standard deviation of errors calculated for every bin.

To quantify learning and to test whether the drug manipulation altered learning, we measured how performance changed across multiple time scales. Rapid trial-by-trial performance changes were quantified via Session (training, follow-up) × Angle (0°, 30°…165°) × Trial (1…8) × Drug (levodopa, placebo) ANOVAs run on the first eight trials of each aiming block. (2) Bin-by-bin and session-by-session performance changes were quantified via Session (training, follow-up) × Bin (1…10) × Angle (0°, 30°…165°) × Drug (levodopa, placebo) ANOVAs run on bin-averaged data (1 bin = mean of eight trials). Across all analyses, we entered three covariates of no interest, as follows: (1) working memory capacity (estimated via memory updating scores), (2) impulsivity (estimated via BIS total scores), and (3) biological sex. These covariates were considered because working memory (Cools et al., 2008), impulsivity (Cools et al., 2007), and sex (Becker, 1999) can affect responsivity to dopamine drug manipulations. Two participants from the levodopa condition did not complete the 165° block due to experimenter error: we included all other data from these participants in all analyses.

We made inferences using Bayesian statistics, as this allowed us to quantify evidence for the test hypothesis, as well as evidence for the null hypothesis. Inclusion Bayes factors (BFinclusion, BF_incl_) were determined to estimate the strength of evidence in favor of including an effect relative to models stripped of that effect. Exclusion Bayes factors (BFexclusion, BF_excl_) were determined to estimate the strength of evidence in favor of excluding an effect relative to models stripped of that effect. In post hoc tests, where evidence for the null hypothesis is reported as BF_01_, and evidence for the test hypothesis is reported as BF_10_, the posterior odds were corrected for multiple testing by fixing to 0.5 the prior probability that the null hypothesis holds across all comparisons (Westfall et al., 1997). Jeffreys's evidence categories for interpretation (Wetzels et al., 2011) were taken as the standard for evaluation of the reported Bayes factors. Specifically, Bayes factors of 1–3 were interpreted as anecdotal, 3–10 as moderate, and >10 as strong evidence for the test hypothesis, whereas Bayes factors of 0.33 or less was taken as evidence for the null hypothesis. Where appropriate, Cohen's *d* values were used to quantify effect sizes and were reported with 95% confidence intervals of the effect size, as estimated using the webapp estimation statistics (Ho et al., 2019). Bayesian analyses were conducted using JAMOVI version 2.3.28. As JAMOVI does not provide covariate-adjusted estimated marginal means, these were obtained using frequentist ANOVAs using SPSS (version 28.0.1.0), for reporting in the figures. We also make our data available at https://osf.io/wnfxt/.

## Results

### Control measurements

Pharmacological manipulations of dopamine can elicit undesired side effects such as nausea, resulting in some participants withdrawing from the study (Chen et al., 2020a), and can also change mood state (Vo et al., 2018). None of our participants reported nausea, and all participants completed the study. Levodopa did not alter our participants’ blood pressure or mood, as Time (before drug administration, 1 h after drug administration, 2 h after drug administration) × Drug ANOVAs yielded evidence for the null hypothesis for Time × Drug interactions for blood pressure (diastole, BF_excl _= 6.383; systole, BF_excl _= 5.683) and mood (BF_excl _= 3.681). Heart rate was reduced to a greater extent for participants on placebo (mean reduction in heart rate, 10.211 ± 1.828) than that on levodopa (mean reduction in heart rate, 1.737 ± 1.828), as shown by a Time × Drug interaction (BF_incl _= 6.519).

Participants were not above chance at accurately guessing the drug condition, as shown by Bayesian binomial tests (BF0+ = 4.685).

### Manipulation check

**Figure 2.**
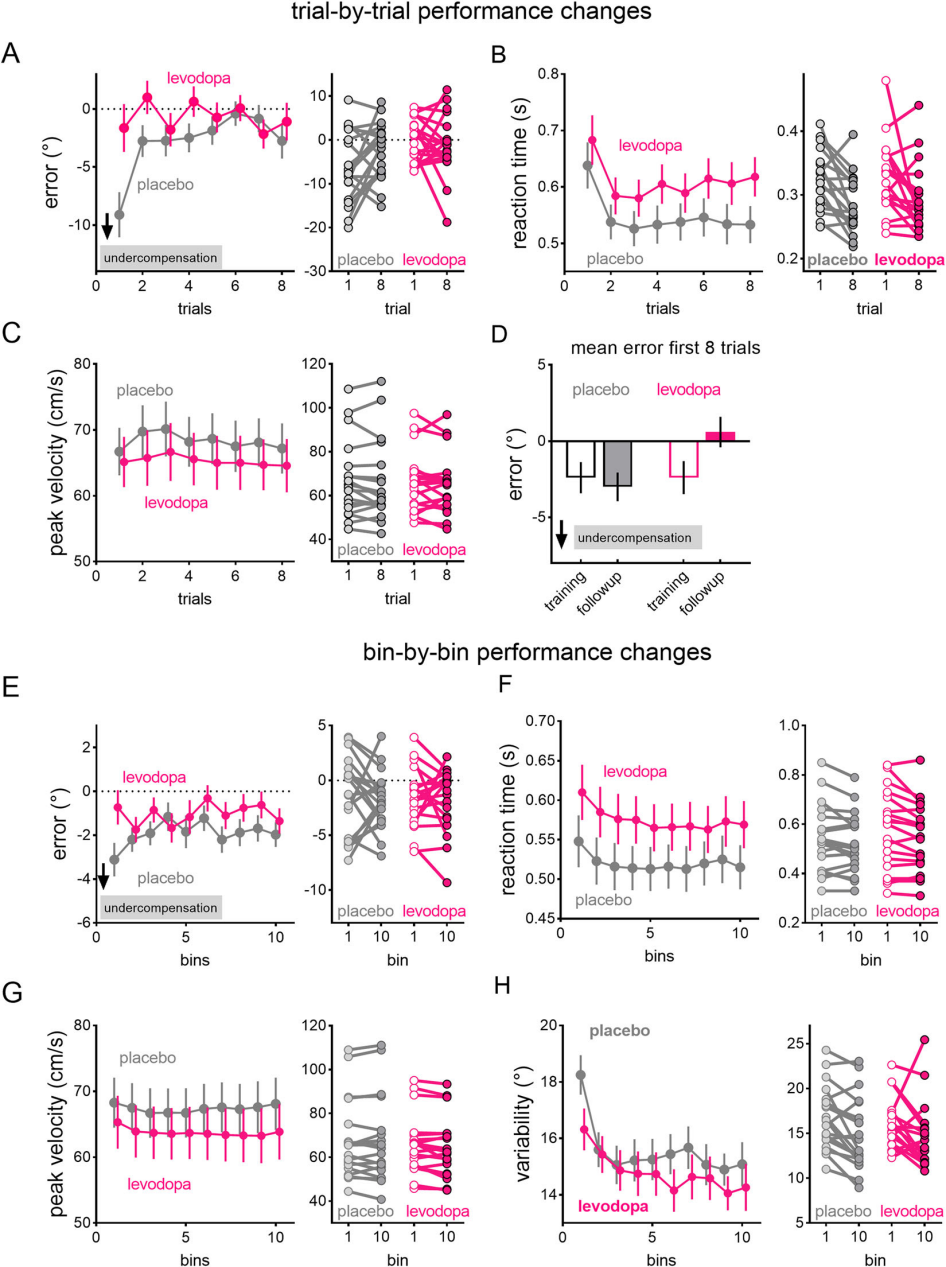
Trial-by-trial performance changes within the first 8 trials (i.e., the first bin) averaged across all aiming angles and across training and follow-up sessions for accuracy (***A***), reaction times (***B***), and peak velocity (***C***). Right panels show changes in individual participant data from trial 1 to trial 8. Trial-wise reductions in reaction times were evident for both the levodopa (pink) and the placebo (gray) groups (***B***, left panel). The levodopa group showed smaller mean errors than the placebo group in the first trial and maintained this level of accuracy across the first eight trials, whereas the placebo group reduced errors across trials. ***D***, Mean error for the first eight trials for the training session compared with the follow-up session. Bin-by-bin performance averaged across the training and follow-up sessions and aiming angles for accuracy (***E***), reaction times (***F***), peak velocity (***G***), and error variability (***H***). Right panels show changes in individual participant data from bin 1 to bin 10. Reaction time and error variability reduced across bins. Values are covariate-adjusted estimated marginal means and standard errors of the mean.

Performance scaled with task difficulty, as larger aiming angles decreased reach accuracy, increased variability and lengthened reaction times (Fig. 2), as supported by main effect of angle for accuracy (BF_incl _= 2.25E + 117), error variability (BF_incl _= ∞) and reaction times (BF_incl _= ∞).

### Trial-by-trial performance changes

We provided knowledge of the task structure and explicit strategies to achieve task goals, which typically leads to fast performance improvements over the first few trials (Mazzoni and Krakauer, 2006).

Rapid improvements in performance were evidenced in reductions in reaction times (Fig. 2*B*; main effect of trial, BF_incl _= 29,887), specifically from the first trial to all subsequent trials (comparisons with first trial, BF_10_ ranging from 6.44 to 1.75E + 8). Reaction times over the first eight trials were overall longer for the levodopa group (BF_10 _= 1,469). However, trial-by-trial reductions in reaction times did not differ between groups, as shown by evidence against the Drug × Trial interaction, BF_excl _= 7,518.80. Peak velocity did not change across trials (evidence against a main effect of trial, BF_excl _= 14.970), regardless of drug condition (evidence against the Drug × Trial interaction, BF_excl _= 1,196.172).

On the first trial, levodopa increased accuracy compared with placebo, as shown by smaller mean errors than placebo [BF_10 _= 4.37; Cohen's *d* = 0.86 (95% CI −1.54, −0.163)]. This high level of accuracy was maintained across trials for the levodopa group, as shown by strong evidence for excluding the main effect of trial (BF_excl _= 4,048.580) in contrast to the placebo group, who reduced error across trials (BF_incl _= 3.856). The levodopa group also showed greater accuracy in the first eight trials at follow-up compared with training (main effect of session, BF_incl _= 11.476; Fig. 2*D*, pink bars), whereas the placebo group showed similar accuracy in the first eight trials for the training and follow-up sessions (main effect of session, BF_excl _= 17.730; Fig. 2*D*, gray bars). Thus, at least for the first eight trials of each aiming block, levodopa resulted in greater session-to-session gains in accuracy compared with placebo.

### Bin-by-bin performance changes

In each session, participants completed 10 bins (80 trials) for each aiming angle. Bin-by-bin performance improvements were evidenced in reduced reaction times and error variability, as shown by strong evidence for the main effect of bin (reaction time, BF_incl _= 2,199.835; variability, BF_incl _= 90,621.064; Figure 2*F*,*H*), while accuracy and peak velocity stayed constant across bins, as shown by strong evidence for excluding the main effect of bin (accuracy, BF_excl _= 90,909.0; peak velocity, BF_excl _= 3.46E + 4).

**Figure 3.**
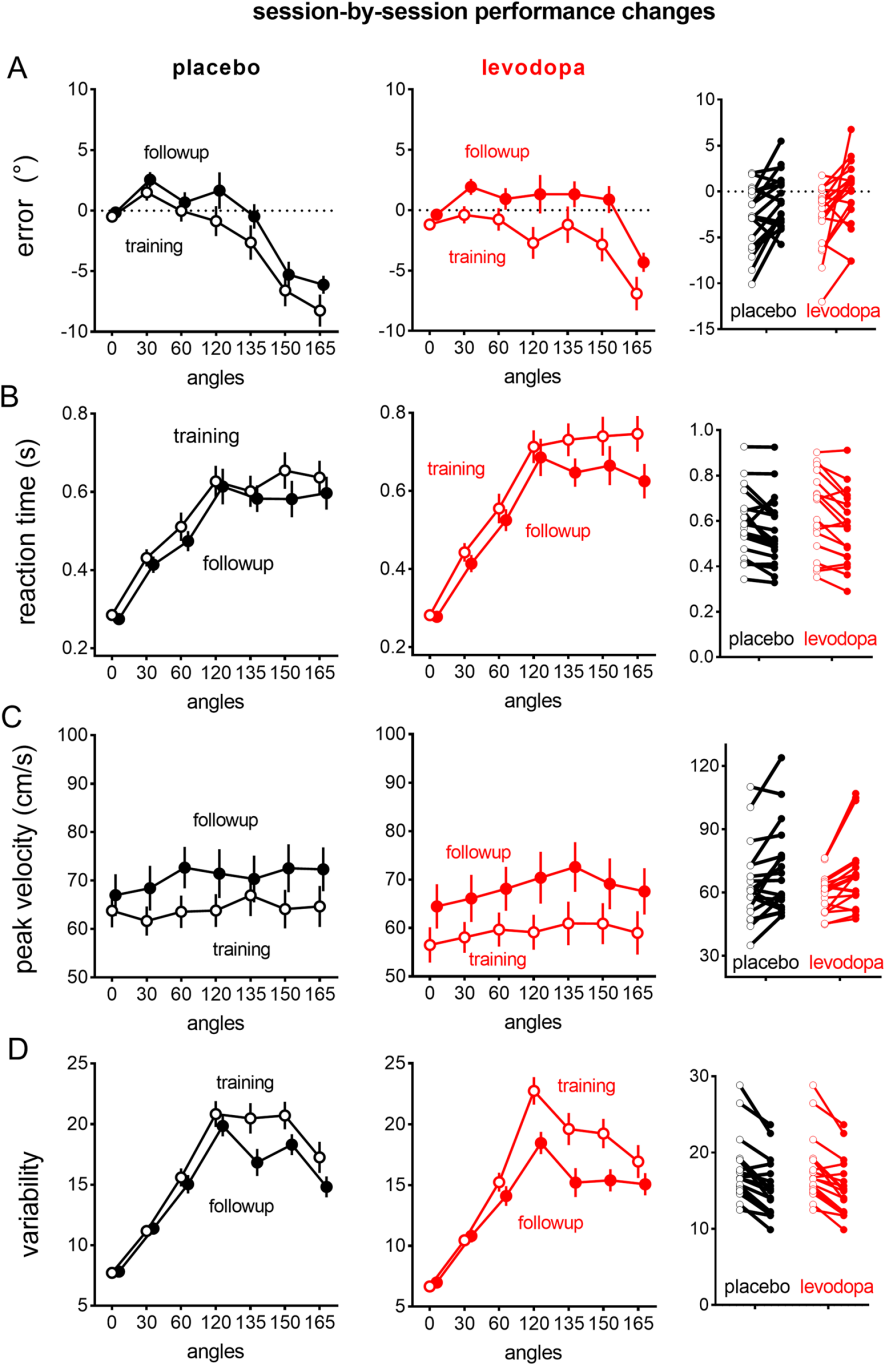
Session-by-session performance improvements in accuracy and the speed of movement planning and movement execution, as evidenced in reduced error, reaction time, and increased peak velocity from training (lighter colors) to follow-up sessions (darker colors), for the levodopa and the placebo group. Values are covariate-adjusted estimated marginal means and standard errors of the mean.

Levodopa did not alter bin-by-bin changes in performance, as shown by strong evidence for excluding Drug × Bin interactions for accuracy (BF_excl _= 5,49,176.79), reaction time (BF_excl _= 3.59E + 6), peak velocity (BF_excl _= 5.57E + 7), and error variability (BF_excl _= 37,037.037) and strong evidence for excluding Drug × Angle × Bin interactions (accuracy, BF_excl _= 6,134.969; reaction time, BF_excl _= 3.45E + 7; peak velocity, BF_excl _= 3.937E + 11; error variability, BF_excl _= 20,040.080). Thus, while levodopa enhanced performance by increasing accuracy and error variability even within the first eight trials (i.e., the first bin), it did not change bin-by-bin performance improvements (Fig. 3).

### Session-by-session performance changes

**Figure 4.**
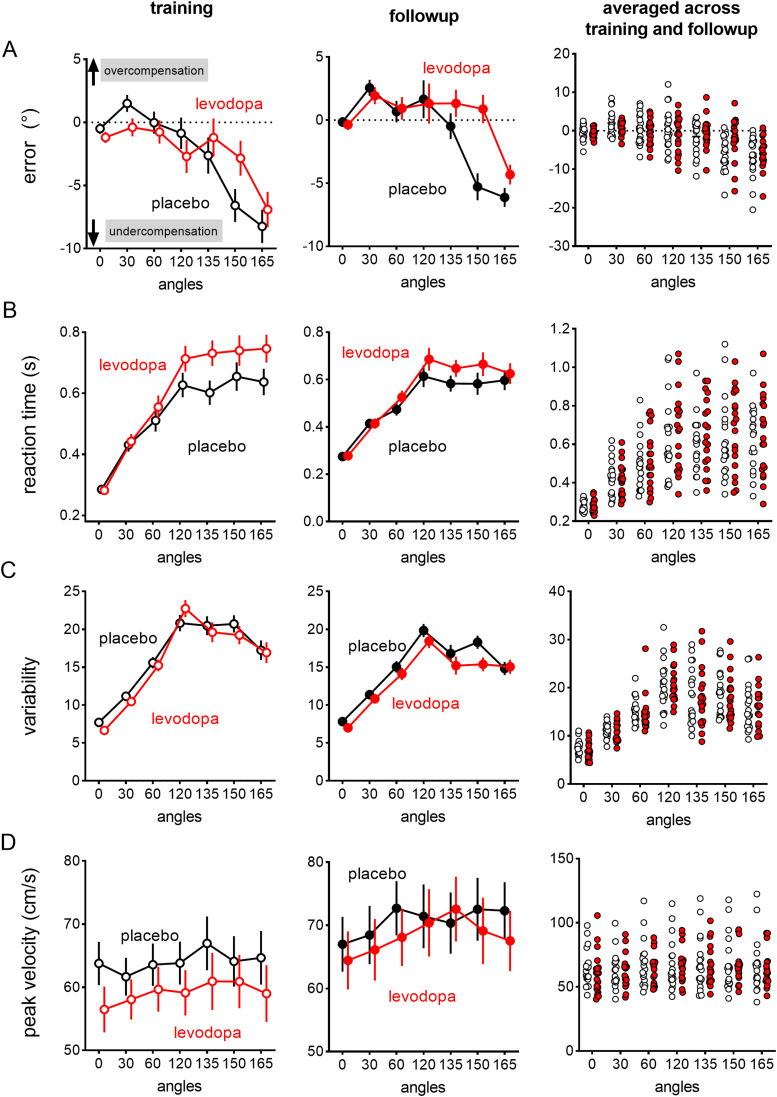
Levodopa modulated accuracy and the speed of movement planning both at training (left panels) and follow-up (middle panels), as shown by effects of levodopa in reducing errors (***A***) while lengthening reaction times (***B***). Levodopa also resulted in slower peak velocity at training (***D***), although this effect was less prominent at follow-up. Values are covariate-adjusted estimated marginal means and standard errors of the mean. Right panels show individual participant data averaged across trial bins across both sessions, for each aiming angle. Values are covariate-adjusted estimated marginal means and standard errors of the mean.

The follow-up session was completed a minimum of 18 h after the training session, which is sufficient to wash out residual effects of levodopa (Contin and Martinelli, 2010). Session-to-session improvements were prominent (Fig. 4), as shown by very strong evidence for main effects of session for accuracy (BF_incl _= 2.41e + 18), reaction time (BF_incl _= 1.86E + 33), peak velocity (BF_incl _= 3.35E + 190), and error variability (BF_incl _= 6.49e + 14).

Levodopa increased session-by-session performance improvements for reaction time for the largest, aiming angles (e.g., 165°), as shown by moderate evidence for Drug × Session × Angle interaction, BF_incl _= 4.175, where the reduction in reaction time from training to follow-up for the largest angle (165°) tended to be larger for the levodopa group [Cohen's *d* = 0.544 (95% CI: 0.279, 0.86)] than the placebo group [Cohen's *d* = 0.267 (95% CI: −0.0937, 0.725)]. For peak velocity, there was strong evidence for a Session × Drug × Angle interaction, BF_incl _= 4,117.00756, where levodopa slowed peak velocity both at training (Drug × Angle interaction, BF_incl _= 454.835) and follow-up (Drug × Angle interaction, BF_incl _= 3.29E + 7), but the effect of levodopa appeared more prominent at training (Fig. 4*D*, left panel) than at follow-up (Fig. 4*D*, middle panel).

While our trial-by-trial analyses showed that levodopa increased offline gains for accuracy for the first bin (first eight trials), this effect was no longer evident across subsequent aiming bins. Levodopa did not modulate session-by-session performance improvements for accuracy and error variability, as shown by moderate evidence for excluding Session × Drug interactions for error (BF_excl _= 4.116) and error variability (BF_excl _= 6.7376), and strong evidence for excluding Session × Drug × Angle interactions for error (BF_excl _= 3.57E + 3) and error variability (BF_excl _= 202.429).

### Levodopa increased accuracy while lengthening reaction times under difficult aiming conditions

Figure 4*A* shows that levodopa administered before training altered aiming performance depending on aiming angle. Aiming accuracy differed depending on aiming angle: for 30°, participants tended to overcompensate (i.e., aim by slightly >30°; Fig. 4*A*), and this overcompensation was somewhat reduced with levodopa. For the larger, more difficult aiming angles (e.g., 150°, 165°), participants tended to undercompensate (aimed by less than the required angle), but this undercompensation was reduced with levodopa. This pattern of results was supported by Angle × Drug interactions for accuracy (BF_incl _= 1.67e + 12). Greater accuracy with levodopa co-occurred with lengthened reaction times, particularly with larger aiming angles, as shown by Angle × Drug interactions for reaction time (BF_incl _= 1.48E + 11; Fig. 4*B*). Notably, the effect of levodopa in increasing accuracy and lengthening reaction times were prominent both at training and follow-up, despite the fact that effects of levodopa consumed at training would have washed out by 8 h after consumption (Contin and Martinelli, 2010), before the start of training.

Thus, levodopa increased accuracy while lengthening reaction times, particularly with the more difficult, larger aiming angles. In a task where success depended on aiming accuracy, increasing dopamine availability via levodopa during training increased the prioritization of accuracy above speed of movement planning, suggesting increased adherence to task goals. This effect persisted at follow-up, after all levodopa effects were washed out. Exogenous dopamine at training thus appeared to result in a learnt, persistent prioritization of task goals, and this occurred in parallel with prominent session-to-session improvements in accuracy and speed of movement planning and movement execution that were mostly unaffected by exogenous dopamine.

## Discussion

Here, we explored the causal role of dopamine in motor learning in neurotypical individuals, by testing how the performance of goal-directed motor strategies was affected by dopamine during training and by testing whether this manipulation altered performance at follow-up, when participants re-encountered the same task. We used a task with varying levels of difficulty and incentivized accurate performance by making task success contingent upon accuracy. Participants were explicitly instructed to use strategies to accurately aim at specified angles from presented targets. Both the task structure and task instructions emphasized accuracy and not speed of movement planning. Here, exogenous dopamine at training increased movement accuracy and lengthened the amount of time taken to plan movements, and this pattern of behavior was evident even within the first eight trials of each aiming angle. This pattern of behavior persisted at follow-up, after all effects of the dopamine medication had been washed out. We posit that this maintained effect reflects some form of learning. While there were marked session-to-session performance improvements, levodopa generally did not increase such offline performance gains, showing only a subtle effect in improving accuracy within the first bin (the first eight trials). Levodopa also did not increase online performance gains. We interpret this result to suggest dopamine altered behavior in an explicit motor learning task, primarily by instilling a learnt propensity to better adhere to task goals (here, to prioritize accuracy over speed of movement planning), not by augmenting the size of online or offline performance gains.

The finding that increasing dopamine availability increased accuracy in an explicit motor learning task that incentivized accuracy supports the view that dopamine influences decisions to engage instrumental motivation in motor learning. These results are consistent with reports that similar exogenous dopamine manipulations can increase deliberative control (Wunderlich et al., 2012; Sharp et al., 2016). Indeed, the focus on explicit motor strategies here might be why dopamine manipulations altered performance here, in contrast to previous null findings (Quattrocchi et al., 2018; Palidis et al., 2021). We provided participants with explicit instructions on how to aim by varying angles from a presented target, where larger aiming angles were associated with larger errors and lengthier reaction times and were more difficult. In contrast, previous work rewarded participants for small, possibly implicit changes in behavior (Palidis et al., 2021), or used traditional paradigms that engaged mutually compensatory implicit and explicit motor learning operations (Quattrocchi et al., 2018). Similarly, pharmacological manipulations of dopamine do not always result in observable effects on learning processes that have been characterized as automatic (Wunderlich et al., 2012; Sharp et al., 2016; Grogan et al., 2017, 2019). Dopamine manipulations often alter the contributions of deliberative, model-based learning processes relative to automatic, model-free learning processes (Wunderlich et al., 2012; Sharp et al., 2016), although exceptions exist (Kroemer et al., 2019). Future studies should dissect how dopamine modulates the relative contributions of deliberative and automatic processes, as automaticity is attained with training during skill learning.

A second feature of this study that differed from previous work (Quattrocchi et al., 2018; Palidis et al., 2021) was the focus on manipulating task success. Instead of manipulating extrinsic rewards (points or money) contingent upon achieving some performance criteria (Galea et al., 2015; Steel et al., 2016; Quattrocchi et al., 2018; Spampinato et al., 2019), we focused on success in achieving the task goal (target-hitting). Participants could only hit targets via accurate aiming, as the targets would move mid-movement by the required aiming angle. Task success thus required accurate performance. Our approach of making task success contingent upon accurate performance might have made our task more sensitive to the effects of exogenous dopamine. In contrast to direct manipulations of task success, which consistently yields clear effects on motor learning (Schaefer et al., 2012; Leow et al., 2018; Kim et al., 2019; Tsay et al., 2022), extrinsic rewards do not always alter motor learning (Steel et al., 2016; Spampinato et al., 2019) or motor performance (Grogan et al., 2020). To investigate how dopamine influences sensitivity to task success and extrinsic rewards, future studies can combine dopamine manipulations with paradigms that dissociate the contributions of task success and extrinsic rewards to performance (Vassiliadis et al., 2021).

Our finding that dopamine increases accuracy under more difficult task conditions is consistent with a view that dopamine guides instrumental motivation in *execution* of effort to attain accurate task performance. This extends previous research in humans, which has largely focused on the role of dopamine in motivation to *select* effortful options, as the majority of previous studies have tested how dopamine manipulations alters decisions to select effortful options in tasks where each decision is only associated with a small or random probability of actually executing that response option (for a review, see Lopez-Gamundi et al., 2021). For example, in effort-based decision-making paradigms, methylphenidate, a dopamine and noradrenaline reuptake inhibitor, increases young adults’ willingness to select more difficult working memory conditions (Westbrook et al., 2019). Dopamine denervation in Parkinson's disease patients results in steeper discounting of effort for reward when choosing between effortful options in comparison with controls (Chong et al., 2015), and this deficit is partially remediated by medications which increase dopamine availability (McGuigan et al., 2019). By showing here that dopamine increased accuracy in a task where participants could not opt out of any trial, but could only choose how well to execute each trial, we infer that dopamine not only alters willingness to select effortful options but also increases willingness to *execute* effort.

How might exogenous dopamine alter the engagement of instrumental motivation during skill acquisition? Influential theories suggest that slow “tonic” dopamine responses signal the background rate of reward and drive motivation (Niv et al., 2007). Fast “phasic” dopamine responses signal unexpected outcomes and help the actor learn to select choices that optimize outcomes (Steinberg et al., 2013). Recent studies however suggest that fast dopamine responses drive both motivation to work for rewards and learning to select options leading to better outcomes (Hamid et al., 2016). Here, we used levodopa, the precursor to dopamine, which increases dopamine availability within the brain. Although we do not fully understand how levodopa affects fast and slow time-scale dopamine responses, animal studies have demonstrated that levodopa increases phasic firing of striatal dopamine cells (Willuhn et al., 2014). We speculate that levodopa employed at training increased accuracy by increasing the amplitude of phasic dopamine cell firing in response to target error feedback. Accuracy improvements that persisted at follow-up might have been associated with training-specific changes in excitatory synaptic transmission in the striatum that were altered by exogenous dopamine at training (Yin et al., 2009).

The increase in accurate aiming with levodopa was accompanied by lengthier deliberation times, suggesting a more cautious mode of response overall—a classic *speed/accuracy tradeoff*. Intriguingly, increases in response caution have not been demonstrated in previous work examining how pharmacological manipulations of dopamine alters decision-making behavior using evidence accumulation modeling (Winkel et al., 2012; Beste et al., 2018; Rawji et al., 2020). In those studies, dopamine manipulations either had no effect on response caution (Winkel et al., 2012), or increased response errors (Huang et al., 2015), or decreased response caution only in the context of proactive inhibition (Rawji et al., 2020) or reinforcement learning task paradigms (Chakroun et al., 2022). These mixed findings might be due to methodological differences, such as the use of different pharmacological agents (e.g., bromocriptine, a dopamine D2 agonist in Winkel et al., 2012; ropinirole, a dopamine D3 agonist in Rawji et al., 2020; and methylphenidate, a dopamine and noradrenaline reuptake inhibitor in Beste et al., 2018) or the use of different tasks [e.g., simple decision-making tasks vs tasks requiring proactive inhibition in Rawji et al. (2020) or tasks requiring learning from reward feedback (Chakroun et al., 2022)]. Here, in a task that rewarded accuracy and not speed, exogenous dopamine resulted in participants trading off speed for accuracy, particularly for difficult task conditions. It will be important for future studies to investigate how dopamine alters performance in contexts where both speed and accuracy are incentivized (Manohar et al., 2015).

Skill learning is accompanied by a violation of the speed accuracy trade-off, where both speed and accuracy are improved (Krakauer et al., 2019). While both speed and accuracy of aiming movements improved markedly at follow-up compared with training, we did not find evidence that dopamine modulated the size of the improvements in speed and accuracy across sessions. This might be due to the timing of the dopamine manipulation. Consistent with our results, animal studies have shown that dopamine agonists and antagonists applied *before* learning have no effect on retention 24 h later but did alter retention when applied 3–6 h *after* initial learning (Bernabeu et al., 1997). Similarly, dopamine medications ingested by Parkinson's disease patients 8–24 h *after* learning improve subsequent recall when tested a day after drug ingestion (Grogan et al., 2015). Dopamine manipulations might thus have modulated offline consolidation of learning only in a critical post-task period: this possibility awaits investigation from future work that tests how the timing of exogenous dopamine affects consolidation.

While we favor the interpretation that dopamine increased motivation to adhere to task goals, increasing accuracy in a task that incentivized accuracy and not speed, we cannot discount the alternative possibility that dopamine increased accuracy by altering valuation of target hits/target misses relative to the cost of time. Indeed, these two interpretations may not be mutually exclusive. Unexpected outcomes such as target misses might evoke phasic responses from midbrain dopamine neurons, evoking a reward prediction error (Schultz et al., 1997). The dopamine manipulation here might have altered the processing of such reward prediction errors, increasing sensitivity to desired outcomes (i.e., target hits) and/or undesired outcomes (target misses) relative to the cost of time, such that actions prioritized accuracy at the cost of time. To disentangle the relative contributions of goal adherence versus target errors to motor performance, future experiments should systematically manipulate higher-order task goals (e.g., differentially incentivize speed vs accuracy) in contexts with or without target hits/misses.

Because we manipulated task success to incentivize accurate performance, we cannot make direct inferences about how dopamine alters “pure” intrinsic motivation to learn and perform motor skills. Increasing evidence demonstrates a key role of dopamine in learning in the absence of extrinsic rewards and in self-evaluating performance without any extrinsic feedback (Ripollés et al., 2018; Duffy et al., 2022). For example, in zebra finches, dopamine neurons show spontaneous activity that correlates with song performance, even in the absence of extrinsic rewards, cues, or external perturbations (Duffy et al., 2022). Similarly, pharmacological manipulation of dopamine in humans can alter learning of novel words in the absence of explicit reward or feedback, and effects remain prominent on a no-drug follow-up session (Ripollés et al., 2018). To test the role of dopamine in intrinsic motivation in motor learning, future studies can combine pharmacological manipulations of dopamine with behavioral paradigms devoid of any form of extrinsic performance feedback, for example, by removing or limiting visual feedback of movement (Vassiliadis et al., 2021) and/or having target appear only transiently at the start of the trial to preclude target hits or target misses (Taylor and Ivry, 2011).

Although we manipulated task success (i.e., target hits in a target-hitting task), our paradigm does not allow us to dissociate if behavior was motivated by avoidance of losses (target misses) or pursuit of rewards (target hits). A large body of knowledge demonstrates that rewards have potent effects in motivating effort (Takikawa et al., 2002; Xu-Wilson et al., 2009; Manohar et al., 2015, 2017; Codol et al., 2019), even when rewards do not depend on how well we perform (Manohar et al., 2017), or when rewards are presented outside our conscious awareness (Pessiglione et al., 2008). Reward effects are evident even in very early visuomotor responses (Carroll et al., 2019; Codol et al., 2023). Reward also makes us more willing to decide to engage costly cognitive resources, such as choosing to perform difficult working memory tasks, switch task sets, or inhibit prepotent responses (Westbrook and Frank, 2018). While less is known about the motivational effects of punishment avoidance, it is clear that avoidance of negative outcomes is a potent driver of behavior (Chen et al., 2020b). While our evidence is broadly supportive of the proposal that dopamine alters motor performance by altering the brain's sensitivity to rewards and increasing instrumental motivation, future studies should disambiguate if the effects shown here resulted from punishment avoidance or reward pursuit.

Our sample was predominantly female. It is clear that there are sex differences in brain dopamine function (Becker, 1999) and responsivity to pharmacological manipulations of dopamine (Dluzen and Ramirez, 1985). Furthermore, although the menstrual cycle phase alters dopamine receptor availability (Czoty et al., 2009), we did not account for individual differences in the menstrual cycle of our female participants. We note that our results were consistent even after removing male participants from our dataset. However, it is unknown if our results will generalize to male participants.

In conclusion, in a task which incentivized accuracy by making task success reliant on accurate motor performance, we find that pharmacologically increasing dopamine availability increased performance accuracy, while concurrently increasing the amount of time used to deliberate and prepare movements, demonstrating a propensity to better adhere to task goals of prioritizing accuracy over speed. This effect of dopamine was sustained even after an overnight delay, long after the effects of exogenous dopamine had washed out. This persistent propensity to prioritize accuracy at follow-up co-occurred with, but was dissociable from, the prominent session-to-session improvements in accuracy and reductions in movement planning time and variability. We interpret the results to suggest that dopamine plays a key role in decisions to engage instrumental motivation to better adhere to task goals, which not only determines the quality of motor performance at initial learning but also influences the quality of future motor performance when the same motor problem is reencountered. While studies in animals and clinical populations have previously demonstrated a role for dopamine in motor learning (Hosp et al., 2011; Isaias et al., 2011), this is, to the best of our knowledge, one of the first evidence for a direct link between dopamine and motor skill learning in neurotypical humans.

## Synthesis

Reviewing Editor: Christophe Bernard, INSERM & Institut de Neurosciences des Systèmes Decisions are customarily a result of the Reviewing Editor and the peer reviewers coming together and discussing their recommendations until a consensus is reached. When revisions are invited, a fact-based synthesis statement explaining their decision and outlining what is needed to prepare a revision will be listed below. The following reviewer(s) agreed to reveal their identity: Samuel McDougle, Richard Ivry. Note: If this manuscript was transferred from JNeurosci and a decision was made to accept the manuscript without peer review, a brief statement to this effect will instead be what is listed below.

Both reviewers agree that the ms has been improved as compared to the JNS submission. They request - although not an absolute necessity - a discussion on the issue.

Some points from previous review: This is a nicely designed and executed exploratory neuropharmacological study, and the results, methods, and motivations are clearly communicated. The double-blinding, open data, and rigorous stats are also very welcome. The long-term memory results of the follow-up session are intriguing. The main concerns with the paper involve the strength of the results and the multiple feasible interpretations.

The drug effects are indeed somewhat circumscribed; they are mainly seen on the first few trials of a bin (why 8 trials in particular?), and the accuracy effects seem to be driven mostly by the first trial in particular. However these are still interesting findings.

Ultimately, the authors argue that DA seemed to change the balance of the SA tradeoff, causing the levodopa group to increase accuracy at the cost of saving time. This is interpreted as an increased adherence to task goals. There are some reasonable alternatives that cannot be ruled out by this study. For instance, the SA tradeoff is sensitive to rewards and to the intrinsic cost of time; could the DA group just value hitting the target more than the placebo group, and thus compute a different cost for taking their time during reach planning?

we would favor discussion about the range of hypotheses rather than the continued focus on one interpretation, "We interpret this result to suggest dopamine altered behaviour in an explicit motor learning task, primarily by instilling a learnt propensity to better adhere to task goals (here, to prioritise accuracy over speed of movement planning)."

In essence the discussion would benefit from a bit more on alternative interpretations and a bit of fleshing out of how open questions could be addressed in future work.

## Author Response

Response to reviewers (round 3).

Dopamine increases accuracy and lengthens deliberation time in explicit motor skill learning

eN-TCFN-0360-23X

Synthesis Statement for Author (Required):

Both reviewers agree that the ms has been improved as compared to the JNS submission. They request - although not an absolute necessity - a discussion on the issue.

Some points from previous review: This is a nicely designed and executed exploratory neuropharmacological study, and the results, methods, and motivations are clearly communicated. The double-blinding, open data, and rigorous stats are also very welcome. The long-term memory results of the follow-up session are intriguing. The main concerns with the paper involve the strength of the results and the multiple feasible interpretations.

The drug effects are indeed somewhat circumscribed; they are mainly seen on the first few trials of a bin (why 8 trials in particular?), and the accuracy effects seem to be driven mostly by the first trial in particular. However these are still interesting findings.

Ultimately, the authors argue that DA seemed to change the balance of the SA tradeoff, causing the levodopa group to increase accuracy at the cost of saving time. This is interpreted as an increased adherence to task goals. There are some reasonable alternatives that cannot be ruled out by this study. For instance, the SA tradeoff is sensitive to rewards and to the intrinsic cost of time; could the DA group just value hitting the target more than the placebo group, and thus compute a different cost for taking their time during reach planning?

we would favor discussion about the range of hypotheses rather than the continued focus on one interpretation, "We interpret this result to suggest dopamine altered behaviour in an explicit motor learning task, primarily by instilling a learnt propensity to better adhere to task goals (here, to prioritise accuracy over speed of movement planning)."

In essence the discussion would benefit from a bit more on alternative interpretations and a bit of fleshing out of how open questions could be addressed in future work.

Response: We have expanded the discussion on alternative interpretations and consideration of how future work could address open questions, as follows.

"Whilst we favour the interpretation that dopamine increased motivation to adhere to task goals, increasing accuracy in a task that incentivised accuracy and not speed, we cannot discount the alternative possibility that dopamine increased accuracy by altering valuation of target-hits/target misses relative to the cost of time. Indeed, these two interpretations may not be mutually exclusive. Unexpected outcomes such as target misses might evoke phasic responses from midbrain dopamine neurons, evoking a reward prediction error (Schultz et al., 1997). The dopamine manipulation here might have altered the processing of such reward prediction errors, increasing sensitivity to desired outcomes (i.e., target hits) and/or undesired outcomes (target-misses) relative to the cost of time, such that actions prioritised accuracy at the cost of time. To disentangle the relative contributions of goal-adherence versus target-errors to motor performance, future experiments should systematically manipulate higher-order task goals (e.g., differentially incentivise speed versus accuracy) in contexts with or without target hits/misses.

Because we manipulated task success to incentivize accurate performance, we cannot make direct inferences about how dopamine alters "pure" intrinsic motivation to learn and perform motor skills. Increasing evidence demonstrates a key role of dopamine in learning in the absence of extrinsic rewards, and in self-evaluating performance without any extrinsic feedback (Ripollés et al., 2018; Duffy et al., 2022). For example, in zebra finches, dopamine neurons show spontaneous activity that correlates with song performance, even in the absence of extrinsic rewards, cues, or external perturbations (Duffy et al., 2022). Similarly, pharmacological manipulation of dopamine in humans can alter learning of novel words in the absence of explicit reward or feedback, and effects remain prominent on a no-drug follow-up session (Ripollés et al., 2018). To test the role of dopamine in intrinsic motivation in motor learning, future studies can combine pharmacological manipulations of dopamine with behavioural paradigms devoid of any form of extrinsic performance feedback, for example by removing or limiting visual feedback of movement (Vassiliadis et al., 2021), and/or having target appear only transiently at the start of the trial to preclude target-hits or target-misses (Taylor and Ivry, 2011).

Although we manipulated task success (i.e., target-hits in a target-hitting task), our paradigm does not allow us to dissociate if behaviour was motivated by avoidance of losses (target-misses) or pursuit of rewards (target-hits). A large body of knowledge demonstrates that rewards have potent effects in motivating effort (Takikawa et al., 2002; Xu-Wilson et al., 2009; Manohar et al., 2015; Manohar et al., 2017; Codol et al., 2019), even when rewards do not depend on how well we perform (Manohar et al., 2017), or when rewards are presented outside our conscious awareness (Pessiglione et al., 2008). Reward effects are evident even in very early visuomotor responses (Carroll et al., 2019; Codol et al., 2023). Reward also makes us more willing to decide to engage costly cognitive resources, such as choosing to perform difficult working memory tasks, switch task sets, or inhibit prepotent responses (Westbrook and Frank, 2018). Whilst less is known about the motivational effects of punishment avoidance, it is clear that avoidance of negative outcomes is a potent driver of behaviour (Chen et al., 2020b). Whilst our evidence is broadly supportive of the proposal that dopamine alters motor performance by altering the brain's sensitivity to rewards and increasing instrumental motivation, future studies should disambiguate if the effects shown here resulted from punishment avoidance or reward pursuit.

## References

[B1] Abe M, Schambra H, Wassermann EM, Luckenbaugh D, Schweighofer N, Cohen LG (2011) Reward improves long-term retention of a motor memory through induction of offline memory gains. Curr Biol 21:557–562. 10.1016/j.cub.2011.02.030 21419628 PMC3075334

[B2] Akam T, Walton ME (2021) What is dopamine doing in model-based reinforcement learning? Curr Opin Behav Sci 38:74–82. 10.1016/j.cobeha.2020.10.010 37082448 PMC7614453

[B3] Albert ST, et al. (2022) Competition between parallel sensorimotor learning systems. Elife 11:e65361. 10.7554/eLife.65361 35225229 PMC9068222

[B4] Barron AB, Sovik E, Cornish JL (2010) The roles of dopamine and related compounds in reward-seeking behavior across animal phyla. Front Behav Neurosci 4:163. 10.3389/fnbeh.2010.00163 21048897 PMC2967375

[B5] Becker JB (1999) Gender differences in dopaminergic function in striatum and nucleus accumbens. Pharmacol Biochem Behav 64:803–812. 10.1016/S0091-3057(99)00168-9 10593204

[B6] Bernabeu R, Bevilaqua L, Ardenghi P, Bromberg E, Schmitz P, Bianchin M, Izquierdo I, Medina JH (1997) Involvement of hippocampal cAMP/cAMP-dependent protein kinase signaling pathways in a late memory consolidation phase of aversively motivated learning in rats. Proc Natl Acad Sci U S A 94:7041–7046. 10.1073/pnas.94.13.7041 9192688 PMC21281

[B7] Beste C, Adelhofer N, Gohil K, Passow S, Roessner V, Li SC (2018) Dopamine modulates the efficiency of sensory evidence accumulation during perceptual decision making. Int J Neuropsychopharmacol 21:649–655. 10.1093/ijnp/pyy019 29618012 PMC6030879

[B8] Bhat RB, Sanes JN (1998) Cognitive channels computing action distance and direction. J Neurosci 18:7566–7580. 10.1523/JNEUROSCI.18-18-07566.1998 9736674 PMC6793264

[B9] Bond A, Lader M (1974) The use of analogue scales in rating subjective feelings. Br J Health Psychol 47:211–218. 10.1111/j.2044-8341.1974.tb02285.x

[B10] Broadway JM, Frank MJ, Cavanagh JF (2018) Dopamine D2 agonist affects visuospatial working memory distractor interference depending on individual differences in baseline working memory span. Cogn Affect Behav Neurosci 18:509–520. 10.3758/s13415-018-0584-6 29569219 PMC6686845

[B11] Buckholtz JW, et al. (2010) Dopaminergic network differences in human impulsivity. Science 329:532. 10.1126/science.1185778 20671181 PMC3161413

[B12] Carroll TJ, McNamee D, Ingram JN, Wolpert DM (2019) Rapid visuomotor responses reflect value-based decisions. J Neurosci 39:3906–3920. 10.1523/JNEUROSCI.1934-18.2019 30850511 PMC6520503

[B13] Chakroun K, Wiehler A, Wagner B, Mathar D, Ganzer F, vanEimeren T, Sommer T, Peters J (2022) Dopamine regulates decision thresholds in human reinforcement learning. bioRxiv:2022.2009. 2029.509499.

[B14] Chen PS, Jamil A, Liu LC, Wei SY, Tseng HH, Nitsche MA, Kuo MF (2020a) Nonlinear effects of dopamine D1 receptor activation on visuomotor coordination task performance. Cereb Cortex 30:5346–5355. 10.1093/cercor/bhaa116 32483622

[B15] Chen X, Voets S, Jenkinson N, Galea JM (2020b) Dopamine-dependent loss aversion during effort-based decision-making. J Neurosci 40:661–670. 10.1523/JNEUROSCI.1760-19.2019 31727795 PMC6961986

[B16] Chong TT, Bonnelle V, Manohar S, Veromann KR, Muhammed K, Tofaris GK, Hu M, Husain M (2015) Dopamine enhances willingness to exert effort for reward in Parkinson's disease. Cortex 69:40–46. 10.1016/j.cortex.2015.04.003 25967086 PMC4533227

[B17] Clatworthy PL, et al. (2009) Dopamine release in dissociable striatal subregions predicts the different effects of oral methylphenidate on reversal learning and spatial working memory. J Neurosci 29:4690–4696. 10.1523/JNEUROSCI.3266-08.2009 19369539 PMC6665353

[B18] Codol O, Holland PJ, Galea JM (2018) The relationship between reinforcement and explicit control during visuomotor adaptation. Sci Rep 8:9121. 10.1038/s41598-018-27378-1 29904096 PMC6002524

[B19] Codol O, Holland PJ, Manohar SG, Galea JM (2019) Reward-based improvements in motor control are driven by multiple error-reducing mechanisms. J Neurosci 40:3604–3620. 10.1523/JNEUROSCI.2646-19.2020 PMC7189755 32234779

[B20] Codol O, Kashefi M, Forgaard CJ, Galea JM, Pruszynski JA, Gribble PL (2023) Sensorimotor feedback loops are selectively sensitive to reward. Elife 12:e81325. 10.7554/eLife.81325 36637162 PMC9910828

[B21] Contin M, Martinelli P (2010) Pharmacokinetics of levodopa. J Neurol 257:253–261. 10.1007/s00415-010-5728-8 21080186

[B22] Cools R, Gibbs SE, Miyakawa A, Jagust W, D'Esposito M (2008) Working memory capacity predicts dopamine synthesis capacity in the human striatum. J Neurosci 28:1208–1212. 10.1523/JNEUROSCI.4475-07.2008 18234898 PMC6671420

[B23] Cools R, Sheridan M, Jacobs E, D'Esposito M (2007) Impulsive personality predicts dopamine-dependent changes in frontostriatal activity during component processes of working memory. J Neurosci 27:5506–5514. 10.1523/JNEUROSCI.0601-07.2007 17507572 PMC6672352

[B24] Cools R, Tichelaar JG, Helmich RC, Bloem BR, Esselink RA, Smulders K, Timmer MH (2022) Role of dopamine and clinical heterogeneity in cognitive dysfunction in Parkinson's disease. Prog Brain Res 269:309–343. 10.1016/bs.pbr.2022.01.012 35248200

[B25] Czoty PW, Riddick NV, Gage HD, Sandridge M, Nader SH, Garg S, Bounds M, Garg PK, Nader MA (2009) Effect of menstrual cycle phase on dopamine D2 receptor availability in female cynomolgus monkeys. Neuropsychopharmacology 34:548–554. 10.1038/npp.2008.3 18256593

[B26] Dluzen D, Ramirez VD (1985) In vitro dopamine release from the rat striatum: diurnal rhythm and its modification by the estrous cycle. Neuroendocrinology 41:97–100. 10.1159/000124160 4047334

[B27] Duffy A, Latimer KW, Goldberg JH, Fairhall AL, Gadagkar V (2022) Dopamine neurons evaluate natural fluctuations in performance quality. Cell Rep 38:110574. 10.1016/j.celrep.2022.110574 35354031 PMC9013488

[B28] Fallon SJ, Kienast A, Muhammed K, Ang YS, Manohar SG, Husain M (2019) Dopamine D2 receptor stimulation modulates the balance between ignoring and updating according to baseline working memory ability. J Psychopharmacol 33:1254–1263. 10.1177/0269881119872190 31526206

[B29] Floel A, Garraux G, Xu B, Breitenstein C, Knecht S, Herscovitch P, Cohen LG (2008) Levodopa increases memory encoding and dopamine release in the striatum in the elderly. Neurobiol Aging 29:267–279. 10.1016/j.neurobiolaging.2006.10.009 17098331 PMC2323457

[B30] Flöel A, Rösser N, Michka O, Knecht S, Breitenstein C (2008) Noninvasive brain stimulation improves language learning. J Cogn Neurosci 20:1415–1422. 10.1162/jocn.2008.20098 18303984

[B31] Galea JM, Mallia E, Rothwell J, Diedrichsen J (2015) The dissociable effects of punishment and reward on motor learning. Nat Neurosci 18:597–602. 10.1038/nn.3956 25706473

[B32] Georgopoulos AP, Massey JT (1987) Cognitive spatial-motor processes. 1. The making of movements at various angles from a stimulus direction. Exp Brain Res 65:361–370. 10.1007/BF00236309 3556464

[B33] Grogan J, Bogacz R, Tsivos D, Whone A, Coulthard E (2015) Dopamine and consolidation of episodic memory: timing is everything. J Cogn Neurosci 27:2035–2050. 10.1162/jocn_a_00840 26102227 PMC4880040

[B34] Grogan JP, Isotalus HK, Howat A, Irigoras Izagirre N, Knight LE, Coulthard EJ (2019) Levodopa does not affect expression of reinforcement learning in older adults. Sci Rep 9:6349. 10.1038/s41598-019-42904-5 31015587 PMC6478852

[B35] Grogan JP, Sandhu TR, Hu MT, Manohar SG (2020) Dopamine promotes instrumental motivation, but reduces reward-related vigour. Elife 9:e58321. 10.7554/eLife.58321 33001026 PMC7599069

[B36] Grogan JP, Tsivos D, Smith L, Knight BE, Bogacz R, Whone A, Coulthard EJ (2017) Effects of dopamine on reinforcement learning and consolidation in Parkinson's disease. Elife 6:e26801. 10.7554/eLife.26801 28691905 PMC5531832

[B37] Hamid AA, Pettibone JR, Mabrouk OS, Hetrick VL, Schmidt R, Vander Weele CM, Kennedy RT, Aragona BJ, Berke JD (2016) Mesolimbic dopamine signals the value of work. Nat Neurosci 19:117–126. 10.1038/nn.4173 26595651 PMC4696912

[B38] Ho J, Tumkaya T, Aryal S, Choi H, Claridge-Chang A (2019) Moving beyond P values: data analysis with estimation graphics. Nat Methods 16:565–566. 10.1038/s41592-019-0470-3 31217592

[B39] Holland P, Codol O, Galea JM (2018) Contribution of explicit processes to reinforcement-based motor learning. J Neurophysiol 119:2241–2255. 10.1152/jn.00901.2017 29537918 PMC6032115

[B40] Hosp JA, Pekanovic A, Rioult-Pedotti MS, Luft AR (2011) Dopaminergic projections from midbrain to primary motor cortex mediate motor skill learning. J Neurosci 31:2481–2487. 10.1523/JNEUROSCI.5411-10.2011 21325515 PMC6623715

[B41] Howard IS, Ingram JN, Wolpert DM (2009) A modular planar robotic manipulandum with end-point torque control. J Neurosci Methods 181:199–211. 10.1016/j.jneumeth.2009.05.005 19450621

[B42] Huang YT, Georgiev D, Foltynie T, Limousin P, Speekenbrink M, Jahanshahi M (2015) Different effects of dopaminergic medication on perceptual decision-making in Parkinson's disease as a function of task difficulty and speed-accuracy instructions. Neuropsychologia 75:577–587. 10.1016/j.neuropsychologia.2015.07.012 26184442

[B43] Isaias IU, Moisello C, Marotta G, Schiavella M, Canesi M, Perfetti B, Cavallari P, Pezzoli G, Ghilardi MF (2011) Dopaminergic striatal innervation predicts interlimb transfer of a visuomotor skill. J Neurosci 31:14458–14462. 10.1523/JNEUROSCI.3583-11.2011 21994362 PMC3212401

[B44] Khan ZU, Gutierrez A, Martin R, Penafiel A, Rivera A, De La Calle A (1998) Differential regional and cellular distribution of dopamine D2-like receptors: an immunocytochemical study of subtype-specific antibodies in rat and human brain. J Comp Neurol 402:353–371. 10.1002/(SICI)1096-9861(19981221)402:3<353::AID-CNE5>3.0.CO;2-4 9853904

[B45] Kim HE, Parvin DE, Ivry RB (2019) The influence of task outcome on implicit motor learning. Elife 8:363606. 10.7554/eLife.39882 PMC6488295 31033439

[B46] Krakauer JW, Hadjiosif AM, Xu J, Wong AL, Haith AM (2019) Motor learning. Compr Physiol 9:613–663. 10.1002/cphy.c170043 30873583

[B47] Kroemer NB, Lee Y, Pooseh S, Eppinger B, Goschke T, Smolka MN (2019) L-DOPA reduces model-free control of behavior by attenuating the transfer of value to action. Neuroimage 186:113–125. 10.1016/j.neuroimage.2018.10.075 30381245

[B48] Kwak Y, Muller ML, Bohnen NI, Dayalu P, Seidler RD (2010) Effect of dopaminergic medications on the time course of explicit motor sequence learning in Parkinson's disease. J Neurophysiol 103:942–949. 10.1152/jn.00197.2009 20018839

[B49] Leow LA, Marinovic W, de Rugy A, Carroll TJ (2018) Task errors contribute to implicit aftereffects in sensorimotor adaptation. Eur J Neurosci 48:3397–3409. 10.1111/ejn.14213 30339299

[B50] Leow LA, Marinovic W, de Rugy A, Carroll TJ (2020) Task errors drive memories that improve sensorimotor adaptation. J Neurosci 40:3075–3088. 10.1523/JNEUROSCI.1506-19.2020 32029533 PMC7141883

[B51] Leow LA, Tresilian JR, Uchida A, Koester D, Spingler T, Riek S, Marinovic W (2021) Acoustic stimulation increases implicit adaptation in sensorimotor adaptation. Eur J Neurosci 54:5047–5062. 10.1111/ejn.15317 34021941

[B52] Lewandowsky S, Oberauer K, Yang LX, Ecker UK (2010) A working memory test battery for MATLAB. Behav Res Methods 42:571–585. 10.3758/BRM.42.2.571 20479189

[B53] Lopez-Gamundi P, Yao YW, Chong TT, Heekeren HR, Mas-Herrero E, Marco-Pallares J (2021) The neural basis of effort valuation: a meta-analysis of functional magnetic resonance imaging studies. Neurosci Biobehav Rev 131:1275–1287. 10.1016/j.neubiorev.2021.10.024 34710515

[B54] Madelain L, Paeye C, Wallman J (2011) Modification of saccadic gain by reinforcement. J Neurophysiol 106:219–232. 10.1152/jn.01094.2009 21525366 PMC3129734

[B55] Manohar SG, Chong TT, Apps MA, Batla A, Stamelou M, Jarman PR, Bhatia KP, Husain M (2015) Reward pays the cost of noise reduction in motor and cognitive control. Curr Biol 25:1707–1716. 10.1016/j.cub.2015.05.038 26096975 PMC4557747

[B56] Manohar SG, Finzi RD, Drew D, Husain M (2017) Distinct motivational effects of contingent and noncontingent rewards. Psychol Sci 28:1016–1026. 10.1177/0956797617693326 28488927 PMC5510684

[B57] Mazzoni P, Krakauer JW (2006) An implicit plan overrides an explicit strategy during visuomotor adaptation. J Neurosci 26:3642–3645. 10.1523/JNEUROSCI.5317-05.2006 16597717 PMC6674132

[B58] McGuigan S, Zhou SH, Brosnan MB, Thyagarajan D, Bellgrove MA, Chong TT (2019) Dopamine restores cognitive motivation in Parkinson's disease. Brain 142:719–732. 10.1093/brain/awy341 30689734

[B59] McNeely ME, Earhart GM (2012) Lack of short-term effectiveness of rotating treadmill training on turning in people with mild-to-moderate Parkinson's disease and healthy older adults: a randomized, controlled study. Parkinsons Dis 2012:623985. 10.1155/2012/623985 22191073 PMC3236457

[B60] Neely KA, Heath M (2010) Visuomotor mental rotation: reaction time is determined by the complexity of the sensorimotor transformations mediating the response. Brain Res 1366:129–140. 10.1016/j.brainres.2010.09.096 20920488

[B61] Niv Y, Daw ND, Joel D, Dayan P (2007) Tonic dopamine: opportunity costs and the control of response vigor. Psychopharmacology 191:507–520. 10.1007/s00213-006-0502-4 17031711

[B62] Palidis DJ, McGregor HR, Vo A, MacDonald PA, Gribble PL (2021) Null effects of levodopa on reward- and error-based motor adaptation, savings, and anterograde interference. J Neurophysiol 126:47–67. 10.1152/jn.00696.2020 34038228

[B63] Pellizzer G, Georgopoulos AP (1993) Common processing constraints for visuomotor and visual mental rotations. Exp Brain Res 93:165–172. 10.1007/BF00227791 8467886

[B64] Pessiglione M, Petrovic P, Daunizeau J, Palminteri S, Dolan RJ, Frith CD (2008) Subliminal instrumental conditioning demonstrated in the human brain. Neuron 59:561–567. 10.1016/j.neuron.2008.07.005 18760693 PMC2572733

[B65] Quattrocchi G, Monaco J, Ho A, Irmen F, Strube W, Ruge D, Bestmann S, Galea JM (2018) Pharmacological dopamine manipulation does not alter reward-based improvements in memory retention during a visuomotor adaptation task. eNeuro 5:1–12. 10.1523/ENEURO.0453-17.2018 PMC6051592 30027109

[B66] Rawji V, Rocchi L, Foltynie T, Rothwell JC, Jahanshahi M (2020) Ropinirole, a dopamine agonist with high D(3) affinity, reduces proactive inhibition: a double-blind, placebo-controlled study in healthy adults. Neuropharmacology 179:108278. 10.1016/j.neuropharm.2020.108278 32827517 PMC7575901

[B67] Ripollés P, Ferreri L, Mas-Herrero E, Alicart H, Gómez-Andrés A, Marco-Pallares J, Antonijoan RM, Noesselt T, Valle M, Riba J (2018) Intrinsically regulated learning is modulated by synaptic dopamine signaling. Elife 7:e38113. 10.7554/eLife.38113 30160651 PMC6133552

[B68] Rosser N, Heuschmann P, Wersching H, Breitenstein C, Knecht S, Floel A (2008) Levodopa improves procedural motor learning in chronic stroke patients. Arch Phys Med Rehabil 89:1633–1641. 10.1016/j.apmr.2008.02.030 18760148

[B69] Schaefer SY, Shelly IL, Thoroughman KA (2012) Beside the point: motor adaptation without feedback-based error correction in task-irrelevant conditions. J Neurophysiol 107:1247–1256. 10.1152/jn.00273.2011 22157120 PMC3289459

[B70] Schultz W, Dayan P, Montague PR (1997) A neural substrate of prediction and reward. Science 275:1593–1599. 10.1126/science.275.5306.1593 9054347

[B71] Shadmehr R, Reppert TR, Summerside EM, Yoon T, Ahmed AA (2019) Movement vigor as a reflection of subjective economic utility. Trends Neurosci 42:323–336. 30878152 10.1016/j.tins.2019.02.003 PMC6486867

[B72] Sharp ME, Foerde K, Daw ND, Shohamy D (2016) Dopamine selectively remediates ‘model-based’ reward learning: a computational approach. Brain 139:355–364. 10.1093/brain/awv347 26685155 PMC5868097

[B73] Spampinato DA, Satar Z, Rothwell JC (2019) Combining reward and M1 transcranial direct current stimulation enhances the retention of newly learnt sensorimotor mappings. Brain Stimul 12:1205–1212. 10.1016/j.brs.2019.05.015 31133478 PMC6709642

[B74] Steel A, Silson EH, Stagg CJ, Baker CI (2016) The impact of reward and punishment on skill learning depends on task demands. Sci Rep 6:36056. 10.1038/srep36056 27786302 PMC5081526

[B75] Steinberg EE, Keiflin R, Boivin JR, Witten IB, Deisseroth K, Janak PH (2013) A causal link between prediction errors, dopamine neurons and learning. Nat Neurosci 16:966–973. 10.1038/nn.3413 23708143 PMC3705924

[B76] Sutton RS, Barto AG (1998) Reinforcement learning: an introduction. Adaptive computation and machine learning. Cambridge, MA: MIT Press.

[B77] Takikawa Y, Kawagoe R, Itoh H, Nakahara H, Hikosaka O (2002) Modulation of saccadic eye movements by predicted reward outcome. Exp Brain Res 142:284–291. 10.1007/s00221-001-0928-1 11807582

[B78] Taylor JA, Ivry RB (2011) Flexible cognitive strategies during motor learning. PLoS Comput Biol 7:e1001096. 10.1371/journal.pcbi.1001096 21390266 PMC3048379

[B79] Tsay JS, Haith AM, Ivry RB, Kim HE (2022) Interactions between sensory prediction error and task error during implicit motor learning. PLoS Comput Biol 18:e1010005. 10.1371/journal.pcbi.1010005 35320276 PMC8979451

[B80] Vassiliadis P, Derosiere G, Dubuc C, Lete A, Crevecoeur F, Hummel FC, Duque J (2021) Reward boosts reinforcement-based motor learning. iScience 24:102821. 10.1016/j.isci.2021.102821 34345810 PMC8319366

[B81] Vo A, Ganjavi H, MacDonald PA (2018) Levodopa has mood-enhancing effects in healthy elderly adults. Int J Geriatr Psychiatry 33:674–675. 10.1002/gps.4824 29498779

[B82] Vo A, Seergobin KN, MacDonald PA (2017) Effects of levodopa on stimulus-response learning versus response selection in healthy young adults. Behav Brain Res 317:553–561. 27743941 10.1016/j.bbr.2016.10.019

[B83] Vo A, Seergobin KN, Morrow SA, MacDonald PA (2016) Levodopa impairs probabilistic reversal learning in healthy young adults. Psychopharmacology 233:2753–2763. 10.1007/s00213-016-4322-x 27241710

[B84] Westbrook A, Frank M (2018) Dopamine and proximity in motivation and cognitive control. Curr Opin Behav Sci 22:28–34. 10.1016/j.cobeha.2017.12.011 29713659 PMC5918294

[B85] Westbrook A, van den Bosch R, Määttä JI, Hofmans L, Papadopetraki D, Cools R, Frank MJ (2019) Dopamine promotes cognitive effort by biasing the benefits versus costs of cognitive work. Science 367:1362–1366. 10.1126/science.aaz5891 PMC7430502 32193325

[B86] Westfall PH, Johnson WO, Utts JM (1997) A Bayesian perspective on the Bonferroni adjustment. Biometrika 84:419–427.

[B87] Wetzels R, Matzke D, Lee MD, Rouder JN, Iverson GJ, Wagenmakers EJ (2011) Statistical evidence in experimental psychology: an empirical comparison using 855 t tests. Perspect Psychol Sci 6:291–298. 10.1177/1745691611406923 26168519

[B88] Willuhn I, Burgeno LM, Groblewski PA, Phillips PE (2014) Excessive cocaine use results from decreased phasic dopamine signaling in the striatum. Nat Neurosci 17:704–709. 10.1038/nn.3694 24705184 PMC4714770

[B89] Winkel J, Van Maanen L, Ratcliff R, Van der Schaaf ME, Van Schouwenburg MR, Cools R, Forstmann BU (2012) Bromocriptine does not alter speed–accuracy tradeoff. Front Neurosci 6:126. 10.3389/fnins.2012.00126 22969702 PMC3430867

[B90] Wunderlich K, Smittenaar P, Dolan RJ (2012) Dopamine enhances model-based over model-free choice behavior. Neuron 75:418–424. 10.1016/j.neuron.2012.03.042 22884326 PMC3417237

[B91] Xu-Wilson M, Zee DS, Shadmehr R (2009) The intrinsic value of visual information affects saccade velocities. Exp Brain Res 196:475–481. 10.1007/s00221-009-1879-1 19526358 PMC2771693

[B92] Yin HH, Mulcare SP, Hilario MR, Clouse E, Holloway T, Davis MI, Hansson AC, Lovinger DM, Costa RM (2009) Dynamic reorganization of striatal circuits during the acquisition and consolidation of a skill. Nat Neurosci 12:333–341. 10.1038/nn.2261 19198605 PMC2774785

